# *USP8* Mutations Associated with Cushing’s Disease Alter Protein Structure Dynamics

**DOI:** 10.3390/ijms252312697

**Published:** 2024-11-26

**Authors:** Natalia Petukhova, Anastasia Poluzerova, Dmitry Bug, Elena Nerubenko, Anna Kostareva, Uliana Tsoy, Renata Dmitrieva

**Affiliations:** 1 Bioinformatics Research Center, Pavlov First Saint Petersburg Medical State University, 197022 Saint Petersburg, Russia; bug.dmitrij@gmail.com; 2 School of Natural Sciences, Tyumen State University, 625003 Tyumen, Russia; anasta874@gmail.com; 3 Almazov National Medical Research Centre, 197341 Saint Petersburg, Russiaanna.kostareva@ki.se (A.K.); tsoy_ua@almazovcentre.ru (U.T.)

**Keywords:** protein modeling, molecular dynamics, docking, USP8 protein, 14-3-3 protein, USP8 mutations, pituitary adenoma, Cushing’s disease

## Abstract

The adenomas in Cushing’s disease frequently exhibit mutations in exon 14, within a binding motif for the regulatory protein 14-3-3 located between the catalytic domain (DUB), responsible for ubiquitin hydrolysis, and the WW-like domain that mediates autoinhibition, resulting in constantly active USP8. The exact molecular mechanism of deubiquitinase activity disruption in Cushing’s disease remains unclear. To address this, Sanger sequencing of *USP8* was performed to identify mutations in corticotropinomas. These mutations were subjected to computational screening, followed by molecular dynamics simulations to assess the structural alterations that might change the biological activity of USP8. Eight different variants of the *USP8* gene were identified both within and outside the “hotspot” region. Six of these had previously been reported in Cushing’s disease, while two were detected for the first time in our patients with CD. One of the two new variants, initially classified as benign during screening, was found in the neighboring SH3 binding motif at a distance of 20 amino acids. This variant demonstrated pathogenicity patterns similar to those of known pathogenic variants. All *USP8* variants identified in our patients caused conformational changes in the USP8 protein in a similar manner. The identified mutations, despite differences in annotation results—including evolutionary conservation assessments, automated predictor data, and variations in localization within exon 14—exhibit similar patterns of protein conformational change. This suggests a pathogenic effect that contributes to the development of CD.

## 1. Introduction

An adrenocorticotropic hormone (ACTH)-secreting pituitary neuroendocrine tumor is a rare pituitary tumor known to cause Cushing’s disease (CD). ACTH overproduction results in an increase in synthesis of glucocorticoids (mainly cortisol) and adrenal androgens by the adrenal cortex and the development of endogenous hypercorticism. The annual incidence of CD is 0.7–2.4 cases per million of population [1,2,3]. Women are affected more often with a ratio of 3–5:1 and the predominant age of diagnosis is between the fourth and fifth decades of life [4,5,6]. This severe endocrine disorder seriously affects patients, leading to life-threatening complications such as obesity, hypertension and cardiovascular disease, diabetes mellitus, dyslipidemia, osteoporosis and fractures, thromboembolic events, infections, mental disorders, sex disorders, and, as a consequence, increased morbidity and mortality compared to the general population [7,8,9,10]. The clinical features and outcomes of CD vary, making it an important concern in neuroendocrinology to identify the factors that determine the biological behavior of corticotropinomas. Since 2015, studies have reported that microadenomas in Cushing’s disease frequently exhibit mutations in exon 14, particularly at positions p.718–720 [11,12,13,14,15,16,17,18,19,20]. The somatic mutations in the *USP8* gene are shown to be associated with Cushing’s disease and are usually detected in 20–60% of adenomas [12,21]. Also, the de novo germline *USP8* variant within the 14-3-3 binding motif was shown to be associated with recurrent CD [22].

The *USP8* gene is located on chromosome 15 and encodes a protein of 1118 amino acids belonging to the USP family. These enzymes play a crucial role in regulating ubiquitin-dependent degradation of membrane proteins by removing ubiquitin tags from substrates. On endosomes, USP8 is involved in regulating the degradation of ubiquitinated receptors, such as EGFR (epidermal growth factor receptor), by deubiquitinating them and preventing lysosomal degradation. By binding with the ESCRT-0 complex (via SH3 motifs), USP8 helps capture and stabilize receptors, preventing their ubiquitin-dependent degradation and retaining them on the membrane for reuse [23,24].

USP8 regulation mechanisms include phosphorylation [25], which facilitates binding with the 14-3-3 protein, proteolytic cleavage [12,26], and autoinhibition [27]. Ubiquitin tags are hydrolyzed intermittently, which helps to regulate the number of receptors on the cell surface, thereby controlling signaling pathways responsible for cell growth, division, and survival [28]. The interaction between USP8 and 14-3-3 proteins is characterized by dynamic behavior, in which binding and dissociation occur depending on the phosphorylation state of USP8. This phosphorylation triggers the binding of 14-3-3 proteins, which temporarily inhibits USP8 activity. However, this interaction is not permanent. As the levels of phosphorylation change, the interaction can cease, allowing USP8 to return to its active state [29].

Mutations in the *USP8* 14-3-3 region result in active USP8 due to disruption of the binding of 14-3-3 proteins to the binding motif. These mutations are located within a region that serves as the binding motif for the regulatory protein 14-3-3 (p.715–720). This motif is situated in an unstructured region between the catalytic domain (DUB) (p.778–1088), responsible for ubiquitin hydrolysis, and the WW-like domain (p.645–684), which mediates autoinhibition.

Amino acid substitutions in pituitary microadenomas associated with Cushing’s disease sometimes occur outside of the phosphorylatable 14-3-3 binding motif, such as p.Gly664Arg [20], which is found within the inhibitory WW-like domain, p.Ala725Asp [11], p.Thr735Ile [11], and p.Asn741Asp [21], which is located in adjacent binding motif (SH3BM/SBM). The latter was found in conjunction with a mutation that falls within the “hotspot” region, specifically the p.Ser719_Gln724delinsLeu mutation.

Given that the 14-3-3 binding motif (715–720 aa) is located within an unstructured region between the WW-like and USP domains, it is possible that binding to the 14-3-3 protein reduces the distance between these domains, thereby promoting autoinhibition. However, conformational changes resulting from mutations are likely to cause alternative interactions both within the protein and with the regulatory 14-3-3 protein, which likely disrupts USP8 inhibition, leading to increased deubiquitinase activity.

The objective of this study is to assess the impact of mutations identified within and outside the 14-3-3 binding motif in the *USP8* gene in adenomas from CD patients on USP8 protein conformation in the context of Cushing’s disease. The comprehensive comparative analysis was performed for mutations previously detected and described in CD patients (p.Ser718Cys, p.Ser718Pro, p.Ser719del, p.Pro720Arg, p.Pro720Gln) and mutations shown to be associated with CD for the first time (p.Thr739Ala and p.Pro720_Asp721delinsArg).

## 2. Results

### 2.1. Genetic Variant Annotation

The annotation of genetic variants identified in patients with Cushing’s disease revealed that most mutations were clinically significant and classified as pathogenic. The p.S718P mutation is one of the most frequently occurring in pituitary adenomas associated with Cushing’s disease, with the p.P720R mutation also being relatively common. Overall, mutations at positions 718 and 720 comprise most of the mutations found in corticotropinomas, whereas the p.S719del mutation is less common [30]. The deletion of amino acids, p.P720_D721delinsR, has not been previously reported; however, deletions of amino acids in the region between 716 and 730, in various combinations, including the deletion of p.720–721, have been documented [11]. The chromatograms of Sanger sequencing showing fragments with mutations for these patients shown in Figure S1. The spectrum of mutations identified in our cohort is presented in Table S1.

**Figure 1:**
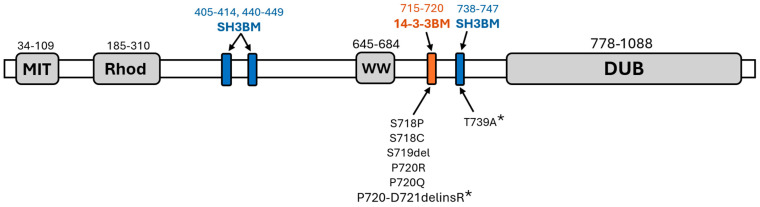
Schematic representation of the protein structure, highlighting functional domains (MIT, Rhod, WW−like, DUB), 14-3-3 and SH3 binding motifs, and the localization and spectrum of detected mutations in our patient cohort. Newly identified mutations (P720_D721delinsR and T739A) are marked with an asterisk (*).

The p.T739A mutation, which has not been previously documented in studies related to Cushing’s disease, is classified as benign in the ClinVar database. According to ClinVar, the evaluation of this variant was conducted in the context of another disease, specifically, hereditary spastic paraplegia, using ACMG-AMP criteria. Therefore, among the mutations identified, two variants (p.P720_D721delinsR and p.T739A) are reported here for the first time in relation to Cushing’s disease. The annotation of all detected variants, along with the mutation locations identified in our study, is illustrated in Figure 1 and presented in Table 1.

### 2.2. The Studied Variants Exhibit Different Levels of Position Conservation

**Figure 2:**
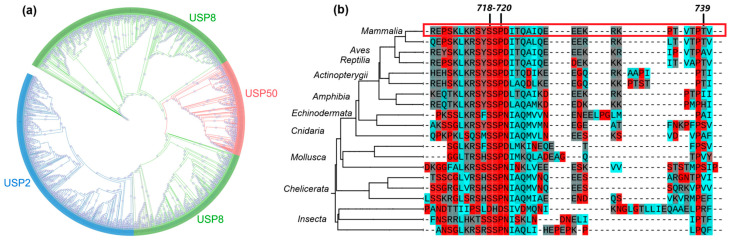
Evolutionary analysis and conservation assessment of positions of interest: (**a**) Phylogenetic tree of USP8, USP50, and USP2. (**b**) Alignment segment of positions 708–740. The alignment uses the “Turn” color scheme from iTOL, where the gradient from red to cyan indicates the propensity to form turns. Red represents the highest propensity, while cyan represents the lowest. Gray shades indicate intermediate propensities.

The evolutionary analysis of USP8 highlights its central role within the family. As shown in Figure 2, the phylogenetic tree presents the green clade (USP8) as the ancestral form of USP50 and USP2. Conservation analysis revealed that most of the amino acid positions we studied are highly conserved in orthologous sequences across various taxa, indicating their potential structural and/or functional importance in the protein. Amino acids at the “hotspots” 718, 719, and 720 are highly conserved among eukaryotes, confirming their likely important role. Position 739 shows variability compared to positions in the 14-3-3 binding motif. Variability, however, does not rule out clinical significance; although the A-to-T substitution has persisted in other species, this may be due to compensatory mutations, differences in the cellular environment, or adaptive mechanisms. Analysis using the “Turn” color scheme showed that the A-to-T substitution at this position demonstrates a difference in turn propensity. The A variant is colored blue, indicating a low propensity for turn formation, while the T variant is gray, reflecting an intermediate propensity. These structural differences suggest that the A-to-T substitution could potentially alter the local conformation of the protein, affecting its overall stability or function, warranting further investigation.

### 2.3. Automated Predictors Did Not Provide a Clear Prediction of the Mutation Effects

Based on the preliminary evaluation of amino acid substitutions p.S718C, p.S718P, p.P720R, p.P720Q, and p.T739A using automated tools for assessing their impact on functionality and stability (Supplementary Figure S2), it was decided to exclude the p.S718P mutation from further analysis. This decision was driven by the consistent prediction of its pathogenicity across various automated tools. Moreover, we identified the p.S718P mutation four times as the sole mutation present in corticotropinomas in our patients with Cushing’s disease. The p.T739A mutation, clinically classified as benign, was initially included as a control for comparison with the wild type.

### 2.4. Analysis of Molecular Dynamics Trajectories: Reduced Catalytic Efficiency of Mutants

**Figure 3:**
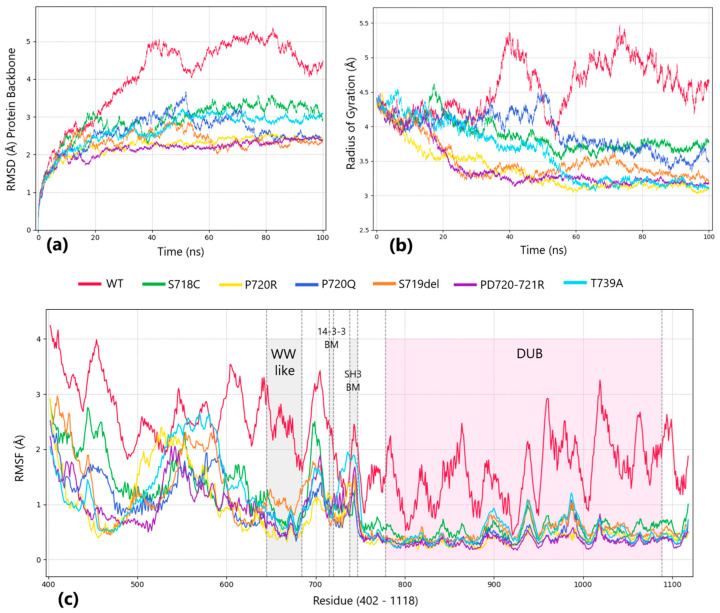
Structural and functional analysis of the USP8 protein using molecular dynamics illustrates the differences in protein behavior between WT and mutants. (**a**) Root mean square deviation (RMSD) of the protein backbone; (**b**) radius of gyration; (**c**) root mean square fluctuation (RMSF) plot comparing WT and mutant USP8 proteins, with the shaded areas highlighting regions of interest, including the catalytic domain (DUB), which demonstrates reduced flexibility in mutants, shaded in pink.

The trajectories were analyzed based on parameters such as root mean square deviation (RMSD), root mean square fluctuation (RMSF), and radius of gyration (Figure 3). The WT demonstrates higher RMSD values and more variable dynamics compared to the mutant trajectories, with the difference between the WT and all mutants being more than 2 Å suggesting their prominent difference in conformational behavior. In contrast, the mutants exhibit a smaller increase in RMSD from their initial coordinates, indicating that the mutations may reduce the protein’s conformational flexibility. The radius of gyration plot also reveals that the wild-type (WT) model is less compact compared to all mutants. These alterations in the compactness and conformation of the protein can significantly impact its functional properties. The deubiquitinase activity of USP8 relies on the ability of its domains to undergo structural adaptation, which is crucial for proper interactions with substrates and regulatory proteins [20]. A decrease in flexibility might impair the ability to hydrolyze ubiquitin bonds, thereby affecting various cellular processes, including protein degradation. The RMSF graph illustrates local structural changes at the level of individual amino acid residues. Particularly, the right side of the plot corresponds to the positions of the catalytic domain (DUB) and is highlighted in pink where the fluctuations for the WT model are significantly higher than those observed in the mutant models. This suggests that the wild type has greater conformational flexibility in the DUB region. The statistical results of MD simulations of USP8 protein models are presented in Table 2.

Moreover, mutant proteins exhibited greater interaction diversity compared to wild-type (WT) models (Table S2). A redistribution of interactions, averaging 4%, was observed between the DUB domain and the region connecting the DUB and WW-like domains in mutants, with overall diversity rising by 12.97%, while the interaction frequency was similar to that of WT proteins (+0.42%). Further analysis identified 15 unique amino acid pairs present across all mutants but absent in WT, all within the DUB domain (Table S3) confirming the changes in bond interplay.

Afterward, we checked the SSE (secondary structure elements) change between WT and mutants during MD simulation, and the additional alpha-helix was detected in all mutant cases in the same region of between 610 and 615 aa. The comparison of secondary structure composition also confirmed structural differences. The helical content was 22.81% in the wild type, while in the mutants, it ranged from 23.74% to 25.46%. This further supports the structural rearrangement in mutant forms and indicates reduced flexibility in the mutant structures compared to the WT. The secondary structure analysis results are provided in Supplementary Materials (Figures S3 and S4).

It is important to note that this study covered a 100-ns timescale for each model, so other contacts may not have been captured within this simulation timeframe. Furthermore, these are intraprotein interactions, while USP8 deubiquitinase actively interacts with various proteins.

## 3. Discussion

It is known that the high enzymatic activity of the USP8 protein promotes the recycling and reuse of the EGFR, thereby enhancing its signaling activity which may facilitate cell proliferation and survival, including that of pituitary cells responsible for ACTH production, and constantly upregulated enzyme activity is considered a part of Cushing’s disease pathogenesis [31,32].

Furthermore, there is evidence that that gain- and loss-of-function of USP8 result in alterations to canonical Wnt signaling because of the shifts in the ubiquitylation-deubiquitylation-recycling process of Fzd receptors, the key regulators of Wnt signaling activity. Thus, if the constantly active USP8-mutant enzyme is expressed in the tumor tissue, an increase in activity of Wnt signaling is expected due to the continuous recycling of Fzd receptors on the tumor cell membrane [33].

The regulation of USP8 activity via interaction between the WW-like and DUB domains is unique to the USP family. The DUB hydrolyzes ubiquitin by capturing it in a binding pocket formed by three main subdomains: fingers, palm, and thumb. Cleavage occurs in the catalytic cleft between the palm and thumb subdomains, where Cys786, His1067, and Asp1084 are responsible for breaking the isopeptide bonds between ubiquitin and its substrates [34]. It was shown that USP8 autoinhibition happens through the binding of the WW-like domain to the DUB, which alters the conformation of the fingers subdomain, blocking ubiquitin access to the active site; attenuated autoinhibition resulting from USP8 mutations could potentially contribute to the development of Cushing’s disease [27].

From the molecular dynamics simulation results, we observed that the catalytic domain undergoes the most significant conformational changes in the mutant proteins. We determined that the mutations have a uniform effect, though we only tested the mutations found in our patients.

Notably, the p.T739A mutation, located twenty amino acids away from the “hotspot” regions, was included in the molecular dynamics analysis as a “benign control”. However, our results indicate that p.T739A exhibits patterns similar to other mutant models. Additionally, the p.T739A mutation is located within the SH3-binding motif (SH3BM)—a motif that interacts with the Hrs-STAM complex (hepatocyte growth factor-regulated tyrosine kinase substrate and signal-transducing adaptor molecule) [24]. This protein complex plays a crucial role in the intracellular sorting and degradation of receptors associated with growth signals and other ligands. The SH3BM motifs include nine amino acids and have the sequence Px(V/I)(D/N)RxxKP [35], with the sequence in the USP8 protein represented as -PTVNRENKP- at positions 738–747. This mutation was found in four out of eighteen samples with Cushing’s disease and showed similar conformational changes in the protein as mutations in the 14-3-3 binding motif, therefore we suggest that this mutation should also be considered as pathological in context of Cushing’s disease.

Our data indicate that USP8 proteins harboring the mutations fold into altered, more compact conformation. These structural changes can significantly affect functional properties, including the deubiquitinase activity of USP8 that relies on the ability of its domains to adapt structurally. This adaptability is crucial for proper interaction with substrates and regulatory proteins. A reduction in flexibility may hinder the hydrolysis of ubiquitin bonds, consequently impacting various cellular processes, including protein degradation [36]. *USP8* variants located within and outside of the “hotspot” region change the protein’s conformation in a way that could lead to the disruption of USP8 autoinhibition due to the lack of interaction with the 14-3-3 protein at the binding motif and/or because of an alternative mode of binding of 14-3-3.

We conducted additional docking analyses on the final frames of the simulation with the 14-3-3β protein across all models (mutants and WT) in order to determine if the mutations in the 14-3-3 binding domain could still bind the regulatory 14-3-3 protein. The results confirmed the absence of binding with 14-3-3BM for all mutants, while also indicating a potential interaction between the 14-3-3 protein and the WW-like domain without phosphorylation involvement. These primary docking studies indicate the altered binding behavior of mutated USP8. We also observed a possible interaction with the unstructured region preceding the WW-like domain. An example of one of the docking complexes is shown in Figure S5. However, we cannot confirm whether this interaction occurs under physiological conditions, as it is known that 14-3-3 typically regulates target proteins through phosphorylated motifs and has specific binding sites with BM. To further clarify the relationship between 14-3-3 and USP8, docking studies should be extended followed by dynamic simulations of the obtained complexes in order to compare interactions with phosphorylated 14-3-3BM as well as the substrate-bound protein form.

## 4. Materials and Methods

### 4.1. Patient Cohort

In this work, we used 35 corticotropinomas from patients with Cushing’s disease to analyze the sequencing data of the *USP8* gene, exon 14, and detected *USP8* variants within and around the 14-3-3 down-regulatory site in 18 samples.

All these patients underwent transsphenoidal surgery between 2012 and 2021 at the Federal Almazov National Medical Research Centre. CD was diagnosed according to the current guidelines [37,38]. Endogenous hypercortisolism was confirmed based on midnight serum or salivary cortisol levels, elevated 24 h urinary free cortisol levels and a lack of cortisol suppression after a low dexamethasone suppression test [37,38]. ACTH-dependent syndrome was diagnosed if the ACTH plasma level was >2 pmol/L [39]. Dynamic pituitary magnetic resonance imaging (MRI) was performed for corticotropinoma visualization. When the MRI pituitary adenoma size was <8 mm, bilateral cavernous and inferior petrosal sinuses sampling was performed as described [40]. The diagnosis of CD was confirmed by histology examination of resected tissue and positive ACTH expression in all samples was shown by immunohistochemistry staining. Patients’ characteristics including morning plasma ACTH and morning serum cortisol levels, as well as clinical outcomes are presented in Table S1. The study was conducted in accordance with the Declaration of Helsinki and was approved by the Institutional Review Board (or Ethics Committee) of Almazov National Medical Research Centre (protocol code 193-4 and date of approval 11 April 2016). All patients signed an institutional-review-board-approved statement of informed consent.

#### Sanger Sequencing of *USP8* Gene

The tumor samples were collected during transsphenoidal surgery. Nine samples were immediately snap-frozen in liquid nitrogen and stored at −80 °C, while another nine samples were stored as paraffin-embedded blocks (FFPE). DNA extraction was performed using Trizol (Thermo Fisher Scientific, Waltham, MA, USA) for native samples and a QIAamp DNA FFPE Advanced Kit (Qiagen, Hilden, Germany) for FFPE blocks. Sanger sequencing of *USP8* exon 14 was performed using a BigDye Terminator Sequencing Kit (Applied Biosystems, Waltham, MA, USAon an ABI PRISM 3100 Genetic Analyzer (Applied Biosystems, Waltham, MA, USA/Hitachi, Tokyo, Japan). The primers were designed using the NCBI Primer Blast forward: 5′-CCCAATCACTGGAACCTTTCG-3′ and reverse: 5′-CCAACTCCCTGACACTAACATAC-3′. The analysis of chromatograms was carried out in the Geneious Prime suite (Biomatters, Auckland, New Zealand) with NCBI Reference *USP8* gene sequence NC_000015.10:50424405-50514421 Homo sapiens chromosome 15, GRCh38.p14 Primary Assembly. The chromatograms of Sanger sequencing showing fragments with mutations are shown in Figure S1.

### 4.2. Prediction of the Effect of Substitutions

For the preliminary assessment of potential functional consequences of genetic alterations in proteins, we used automated prediction tools such as PredictSNP [41], MAPP [42], PhD-SNP [43], PolyPhen-2 [44], SIFT [45], SNAP [46], and AlphaMissense [47]. To predict the effect of these mutations on protein thermodynamic stability, we employed a different set of automated tools including I-Mutant 2.0 [48], MUpro [49], mCSM [50], DUET [51], SDM [52], and PremPS [53]. By aggregating these predictions, we gained preliminary insights into the likely consequences and impact assessments.

### 4.3. Evolutionary Analysis and Conservation Evaluation

A BLAST search in the NCBI database was carried out using default settings, with the query being the sequence of the protein encoded by the *USP8* gene, identified by accession number P40818. Sequence alignment was performed using the MAFFT algorithm [54]. Phylogenetic trees were constructed with the IQ-TREE software package (version 1.6.12) [55], employing bootstrapping with 1000 iterations. The constructed phylogenetic tree enabled the selection of a clade of *USP8* orthologs to assess the conservation of the positions of interest. For tree visualization and annotation, we used the web tool iTOL (https://itol.embl.de/) [56]. Clades were collapsed at the level of 0.05> average branch length to remove highly similar groups, and the aligned sequences were mapped to the tree by matching names and RefSeq numbers.

### 4.4. Protein Structure Modeling

Molecular modeling of the USP8 protein was conducted to prepare a structurally validated model for further studies on the effect of mutations in molecular dynamics simulations. The predicted full-length AlphaFold model (AF-P40818-F1) from the AlphaFold database (https://alphafold.ebi.ac.uk/, accessed on 24 November 2024) [57], covering all amino acid residues from 1 to 1118, was used as the basis for creating the required model. The model was trimmed to residues 402 to 1118, which included the catalytic domain and regions of low structural order potentially involved in protein function regulation. To ensure structural integrity, alignment was carried out using the Prime Maestro 13.4 package (Schrödinger, LLC, New York, NY, USA) with the catalytic domain model in its closed form (PDB ID—2GFO) [34]. Zinc ion was incorporated into the final model. The structural alignment resulted in an alignment score of 0.051 and an RMSD of 1.123 Å, indicating a high-quality match. Comprehensive analysis of all atomic contacts and protein preparation was conducted using MolProbity [58] which validated the model’s suitability for dynamic simulations.

### 4.5. Structural and Functional Analysis of the USP8 Protein Using Molecular Dynamics

First of all, we employed the constructed USP8 model into energy minimization and relaxation using the GROMACS 2023.3 software package [59] for 200 ns to ensure structural stabilization and to enhance model accuracy. The steepest descent algorithm was employed, involving 50,000 nsteps with an energy tolerance (emtol) of 1000.0 kJ/mol.nm and an energy minimization step size (emstep) of 0.01 nm. The simulation used simple point charge water and the AMBER99SB-ILDN force field. The NVT ensemble (constant volume and temperature) maintained the system at 300 K, controlled by a V-rescale thermostat. Hydrogen bond constraints were applied using the LINCS algorithm. The (particle mesh ewald) method handled long-range Coulombic interactions, and the Verlet neighbor list facilitated efficient interatomic interaction calculations. The next stage used the NPT ensemble (constant particles, pressure, and temperature) with the Parrinello–Rahman barostat to maintain pressure. The final productive phase of protein relaxation lasted 200 ns in physiological saline (0.15 M NaCl). All relaxation stages aimed to closely mimic physiological conditions to ensure realistic modeling of USP8 protein behavior at the atomic level.

The final frame of the 200 ns relaxation simulation was chosen as the template for further studies of mutant forms of the USP8 protein model. Relevant mutations were introduced into this relaxed USP8 model using the Maestro Schrödinger 13.4 package. All protein models, including the wild-type and its mutant forms, underwent 100 ns simulations. Molecular dynamics trajectories and interactions were analyzed using the ProLIF package [60] and the MDAnalysis library [61].

To assess the changes induced by the structural rearrangement of the protein, we analyzed intraprotein interactions between the DUB domain and the rest of the protein, as well as interactions within the DUB domain itself. These interactions were categorized into structural segments, allowing for a comparison of altered interaction distributions in mutants with the wild-type form. The data were first calculated separately and then also averaged to quantify the observed differences. In addition, we identified pairs of amino acids that were present in the mutant forms but absent in the wild type. This was achieved by comparing trajectory dataframes of the mutant forms and identifying common amino acid pairs, which were then cross-referenced with those found in the wild-type protein.

A secondary structure analysis was performed using Gromacs, and graphs were generated using the Python packages Pandas and Matplotlib.

## 5. Conclusions

Based on the results from studying the impact of mutations in exon 14 of the *USP8* gene on deubiquitinase regulation, it can be inferred that specific mutations within the unstructured interdomain region at positions p.718–720—including p.Ser718Pro, p.Ser718Cys, p.Pro720Arg, p.Pro720Gln, and p.Ser719del, as well as the previously unreported p.Pro720_Asp721delinsArg and p.Thr739Ala—exhibit pathogenic effects. Despite the biochemical diversity and evolutionary conservation of these variants, all examined mutations appear to alter the regulation of catalytic domain activity, resulting in similar molecular and clinical outcomes. This suggests that mutations in the interdomain region induce an abnormal conformation that differs from the wild-type protein. Moreover, all mutations occurred at the low complexity locus that led to conformation alterations in the whole molecule causing potential dysfunction and dysregulation; similar disease-related effects were demonstrated for other proteins [62,63,64,65].

By analyzing the intramolecular interactions of all our models during molecular dynamics simulations, we observed that the variability of interaction pairs in mutants increased by 12.97%, while the overall frequency of interactions remained comparable to that of WT proteins (+0.42%). Secondary structure analysis also indicated differences, confirming the formation of more rigid proteins in mutants.

We also assume that mutations in the neighboring SH3-binding motif (such as Thr739Ala), located within the same interdomain region, could similarly disrupt the autoinhibition mechanism, thereby increasing DUB activity. This mutation is likely of significant importance in the development of Cushing’s disease pathogenesis, as it was identified in three patients in the absence of other mutations in exon 14. To verify this hypothesis, further research is necessary to explore the interactions between the Hrs-STAM complex and USP8 and to investigate the dynamic behavior of USP8 with all regulatory proteins over time.

## Figures and Tables

**Table 1:** Variants annotation.

Substitution	N of Patients	Codon	Allele	Position GRCh38.p14 chr15	rsID	Clinical Significance
S718P	4	TCC>CCC	T>C	50,490,443	rs672601307	pathogenic
S718C	1	TCC>TGC	C>G	50,490,444	rs672601308	pathogenic
S719del	3	TCC	TCC>del	50,490,446–50,490,448	rs672601306	pathogenic
P720R	2	CCA>CGA	C>G	50,490,450	rs672601311	pathogenic
P720Q	3	CCA>CAA	C>A	50,490,450	-	-
P720_D721delinsR	1	CCAGAT> CGT	Deletion-insertion	50,490,450–50,490,455	-	-
T739A	3	ACA>GCA	A>G	50,490,506	rs11638390	benign
T739A	1	ACA>GCA	A>G	50,490,506	rs11638390	benign
+ S718P		TCC>CCC	T>C	50,490,443	rs672601307	pathogenic

**Table 2:** Statistics of MD simulations for USP8 protein models. The analysis of MD simulations for USP8 protein models, including the wild type (WT) and various mutations is shown. The mean (average) and STD (standard deviation) are provided for three metrics: RMSD backbone (stability), RMSF (flexibility), and RG (radius of gyration, compactness). Lower RMSD and RG values indicate more stable, compact structures, while a higher RMSF suggests greater flexibility.

Model	RMSD Backbone		RMSF		RG	
	Mean	STD	Mean	STD	Mean	STD
WT	4.049	1.062	2.101	0. 777	4.558	0.369
S718C	2.886	0.515	9.774	0.596	3.868	0.219
S719del	2.322	0.324	8.955	0.582	3.506	0.287
P720R	2.233	0.302	7.288	0.551	3.364	0.313
P720Q	2.580	0.478	7.518	0.486	3.920	0.273
P720-D721delinsR	2.140	0.267	6.783	0.458	3.407	0.343
T739A	2.650	0.473	8.995	0.612	3.608	0.428

## Data Availability

The additional materials supporting reported results available upon reasonable request and at https://github.com/anasta874/PC-SFC (accessed on 24 November 2024).

## Supplementary Materials

The following supporting information can be downloaded at: https://www.mdpi.com/article/10.3390/ijms252312697/s1.
