# Supplementary material for: USP8 Mutations Associated with Cushing’s Disease Alter Protein Structure Dynamics
Source: Int J Mol Sci. 2024 Nov 26;25(23):12697. doi: 10.3390/ijms252312697 (PMC11641493; doi:10.3390/ijms252312697)
Supplement: Supplementary file 1 [file ijms-25-12697-s001.zip › ijms-3293972-supplementary.pdf]

Supplementary Material

USP8 mutations associated with Cushing's disease alter protein structure dynamics

Natalia Petukhova [1,*], Anastasia Poluzerova [2], Dmitry Bug [1], Elena Nerubenko [3], Anna Kostareva [3] , Uliana Tsoy [3] and Renata Dmitrieva [3,*]

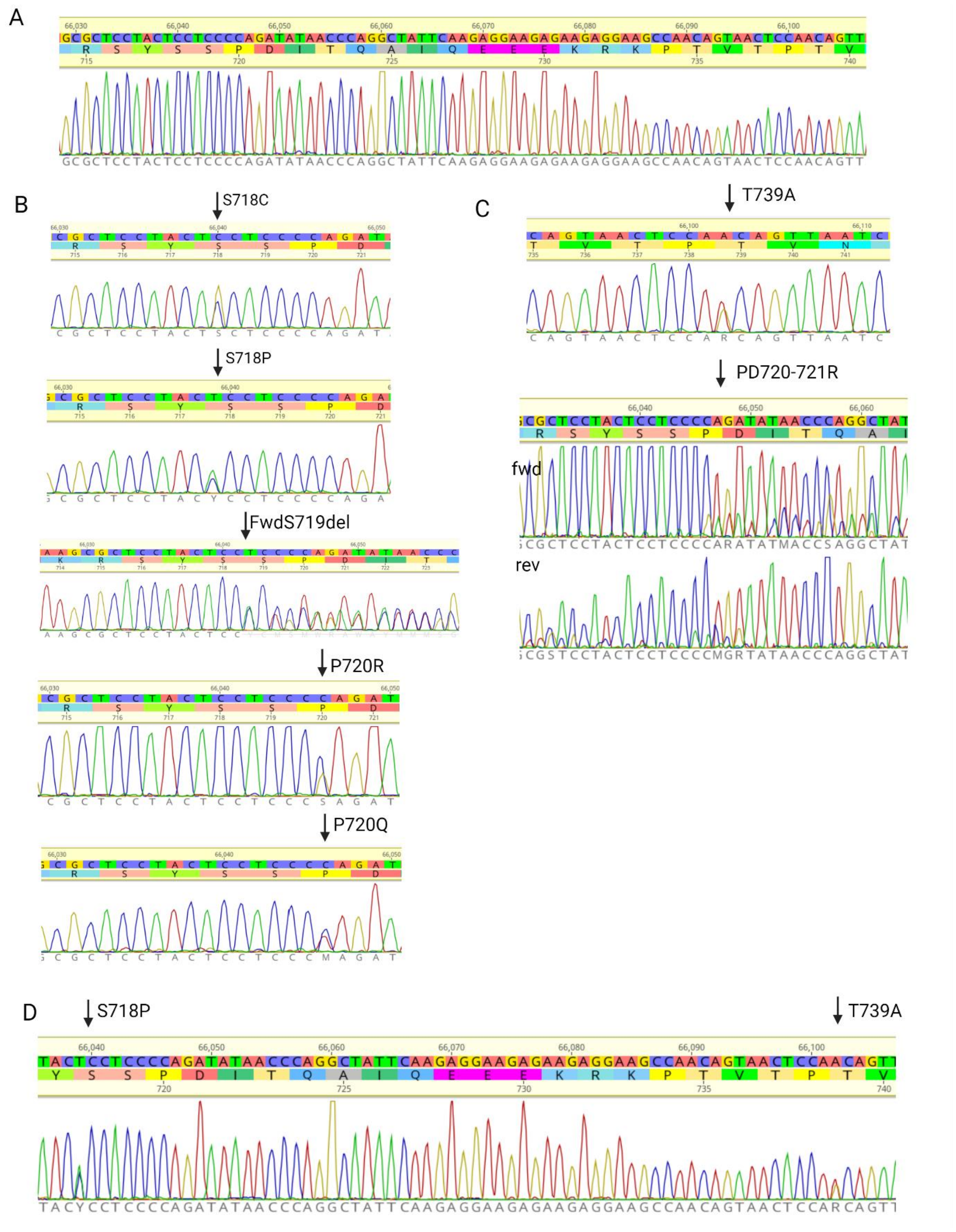

Supplemental Figure 1: Chromatograms of 14 exon USP8 Sanger sequencing showing fragments with mutations for patients. (A) Fragment of 14 exon USP8 gene with wild-type; (B) Known mutations for patients with Cushing's disease (CD); (C) Mutations were not previously described for patients with CD; (D) The patient carrying two kinds of mutation.

№	ID	USP8 mutation status	Sex Female Male	Age, years	The largest adenoma's diameter, mm	Volume, ml	ACTH, pg/ml	Morning serum cortisol, nmol/l	24-h urine cortisol excretion, nmol/l	Ki 67, %	SST2	SST5	Remission of hypercortisolism after TSS, yes/no	Follow-up period, years	Remission of hypercortisolism, follow up, yes/no	Adenoma growth, follow up, yes/no
1	P1	**S718P**	F	66	15	1.41	38.57	696.1	137.88	0.3	>80%	>80%	Yes	3	Yes	No
2	P31	**S718P**	F	30	18	1.45	99.7	749	420.12	1.4	>60%	>90%	Yes	1	Yes	No
3	C112	**S718P**	F	34	12	0.41	97.6	613.8	662.4	3.29	30%	>80%	No	2	No	Yes
4	C61	**S718P**	F	37	15	0.3	83.45	594.2	1540.9	0.4	NA	NA	Yes	2	No	Yes
5	c81	**T739A**	F	67	3	0.003	41.81	735	485	-	NA	NA	Yes	4	Yes	No
6	P122	**T739A**	F	56	4.6	0.016	71.73	896.6	244.26	-	NA	NA	Yes	9	No	Yes
7	C120	**T739A**	F	58	6	0.07	58.8	582.2	156.6	0.4	NA	NA	Yes	8	Yes	No
8	c73	**S718P, T739A**	M	20	8	0.25	78.96	778	630	0.8	NA	NA	Yes	1	Yes	No
9	P20	**FwdS719del**	F	60	12	0.28	23.54	901.1	382	1	5%	>80%	Yes	3	Yes	No
10	c126	**FwdS719del**	F	25	18	1.03	54.71	1094	500	2.84	3%	>60%	No	3	No	Yes
11	P3	**FwdS719del**	F	18	16.7	1.56	41.81	758.6	-	0.6	>60%	>90%	Yes	2	Yes	No
12	P28	**P720Q**	F	39	8.5	0.11	53.47	-	149	5.3	>80%	>60%	Yes	3	No	Yes
13	P22	**P720Q**	F	31	10.5	0.26	120	743.2	1752	-	>50%	>90%	Yes	1	Yes	No
14	P25	**P720Q**	F	26	12	0.31	128.3	448	352.58	4.5	50%	>30%	Yes	1	No	Yes
15	P27	**P720R**	F	36	8.5	0.12	81.2	471.4	892	-	NA	>80%	Yes	1	Yes	No
16	c150	**P720R**	F	37	14	0.43	55	734	708	3.24	60%	>60%	Yes	1	Yes	No
17	29	**S718C**	F	36	5	0.06	78.69	1066	-	-	NA	NA	Yes	2	No	Yes
18	c117	**PD720-721R**	F	38	9	0.1	64.34	535	648.25	0.1	15%	>80%	Yes	8	No	Yes

*

ACTH – adrenocorticotropic hormone, TSS-transsphenoidal surgery, NA- not applicable

Supplemental Table 1: Clinical data and USP8 status in patients enrolled into the study

SNP	PredictSNP	MAPP	PhD-SNP	PolyPhen-2	SIFT	SNAP	Alpha Missense
p.S718C	Pathogenic	Neutral	Pathogenic	Pathogenic	Pathogenic	Neutral	Pathogenic
p.S718P	Pathogenic	Pathogenic	Pathogenic	Pathogenic	Pathogenic	Neutral	Pathogenic
p.P720R	Pathogenic	Neutral	Neutral	Pathogenic	Pathogenic	Pathogenic	Pathogenic
p.P720Q	Neutral	Neutral	Neutral	Pathogenic	Pathogenic	Pathogenic	Pathogenic
p.T739A	Neutral	Neutral	Neutral	Neutral	Neutral	Neutral	Neutral

(a)

SNP	MUpro	mCSM	DUET	SDM	DynaMut	PremPS
p.S718C	Pathogenic	Pathogenic	Pathogenic	Neutral	Neutral	Pathogenic
p.S718P	Pathogenic	Pathogenic	Pathogenic	Pathogenic	Pathogenic	Pathogenic
p.P720R	Pathogenic	Neutral	Neutral	Neutral	Neutral	Pathogenic
p.P720Q	Pathogenic	Pathogenic	Neutral	Neutral	Neutral	Pathogenic
p.T739A	Pathogenic	Pathogenic	Neutral	Neutral	Neutral	Pathogenic

(b)

Supplemental Figure 2: Preliminary evaluation using automated tools. "Pathogenic" and "Neutral" effects are indicated.
(a) Initial assessment of functional impact. The tools in this table are sourced from the PredictSNP web resource and include PredictSNP, MAPP, PhD-SNP, PolyPhen-2, SIFT and SNAP, representing consensus predictions or specific threshold values. The results for Alpha Missense are sourced from the Alpha Missense prediction dataset.
(b) Initial evaluation of effects on thermodynamic stability using tools MUpro, mCSM, DUET, SDM, DynaMut, and PremPS. DUET combines results from mCSM and SDM.

All interactions calculated over a 100 ns simulation (10,000 frames) :

	DUB domain interaction with region (402–644)				DUB domain interaction with WW-Like region (645—684)				DUB domain interaction with interdomain region (685—776)				DUB domain (intradomain interactions) (777 – 1088)				DUB domain interaction with post-DUB region (1089 – 1118)			
	N		*F*		*N*		*F*		*N*		*F*		*N*		*F*		*N*		*F*	
WT	**132**	**0**	**68 326**	**0**	**26**	**0**	**1550**	**0**	**97**	**0**	**133 092**	**0**	**5852**	**0**	**21 576 263**	**0**	**114**	**0**	**310 218**	**0**
S718C	331	**+150.76%**	105 312	**+54.13%**	35	**+34.62%**	10 356	**+568.13%**	92	**-5.15 %**	142 796	**+7.29%**	5998	**+2,49**	21 515 218	**-0.28%**	119	**+4.39%**	290 656	**−6.31%**
P720R	266	**+101.52%**	100 715	**+47.40%**	97	**+273.08%**	30 806	**+1887.48%**	96	**-1.03 %**	154 648	**+16.20%**	6173	**+5,49%**	21 714 144	**+0.64%**	114	**0.0%**	326 208	**+5.15%**
P720Q	200	**+51.52%**	72 389	**+5.95%**	19	**-26.92%**	8 938	**+476.65%**	94	**-3.09%**	151 139	**+13.56%**	6242	**+6.66%**	21 530 820	**-0.21%**	117	**+2.63%**	296 197	**−4.52%**
S719del	377	**+185.61%**	148 440	**+117.25%**	34	**+30.77%**	2 456	**+58.45%**	88	**-9.28%**	148 730	**+11.75%**	6188	**+5.74%**	21 488 159	**-0,41%**	109	**−4.39%**	310 823	**+0.20%**
PD720-721R	212	**+60.61%**	95 516	**+39.79%**	51	**+96.15%**	35 836	**+2212.00%**	86	**-11.34%**	148 263	**+11.40%**	6030	**+3,04%**	21 799 267	**+1,03%**	126	**+10.53%**	272 282	**−12.23%**
T739A	196	**+48.48%**	80 787	**+18.24%**	18	**-30.77%**	3 747	**+141.74%**	104	**+7.22%**	151 238	**+13.63%**	6058	**+3,52%**	21 638 556	**+0.29%**	105	**−7.89%**	307 644	**−0.83%**
Mutants (mean)	264	**+100.0%**	100 526	**+47.13%**	42	**+61.54%**	15 356	**+890.71%**	93	**-4.12%**	149 469	**+12.31%**	6115	**+4,49%**	21 614 361	**+0.18%**	115	**+0.88%**	300 635	**-3.09%**

Interactions exceeding 10% of the simulation time (more than 1,000 frames) :

WT	**18**	**0**	**59520**	**0**	**0**	**0**	**0**	**0**	**23**	**0**	**121028**	**0**	**3056**	**0**	**21 149 564**	**0**	**50**	**0**	**303343**	**0**
S718C	29	**+61.11%**	76028	**+27.74%**	4	-	6769	-	26	**+13.04%**	134922	**+11.48%**	3022	**-1.11%**	21 044 042	**-0.5%**	46	**-8.00%**	279811	**-7.76%**
P720R	26	**+44.44%**	73912	**+24.18%**	11	-	16375	-	29	**+26.09%**	144221	**+19.16%**	3136	**+2.62%**	21 221 172	**+0.34%**	46	**-8.00%**	317227	**+4.58%**
P720Q	22	**+22.22%**	58416	**-1.85%**	3	-	7051	-	30	**+30.43%**	140391	**+16.00%**	3143	**+2.85%**	21 029 046	**-0.57%**	45	**-10.00%**	285720	**-5.81%**
S719del	41	**+127.78%**	115276	**+93.68%**	0	-	0	-	27	**+17.39%**	142359	**+17.62%**	3050	**-0.2%**	20 991 095	**-0.75%**	45	**-10.00%**	305111	**+0.58%**
PD720-721R	23	**+27.78%**	79880	**+34.21%**	9	-	29446	-	33	**+43.48%**	140933	**+16.45%**	3105	**+1.6%**	21 337 546	**+0.89%**	50	**0.0%**	260654	**-14.07%**
T739A	19	**+5.56%**	62545	**+5.08%**	2	-	2706	-	29	**+26.09%**	136026	**+12.39%**	3070	**+0.46%**	21 177 164	**+0.13%**	52	**+4.00%**	300893	**-0.81%**
Mutants (mean)	27	**+50.00%**	77676	**+30.50%**	5	-	10391	-	29	**+26.09%**	139809	**+15.52%**	3088	**+1.05%**	21 101 323	**-0.23%**	47	**-6.00%**	291569	**-3.88%**

Interactions exceeding 30% of the simulation time (more than 3,000 frames) :

WT	**11**	**0**	**48827**	**0**	**0**	**0**	**0**	**0**	**16**	**0**	**106809**	**0**	**2396**	**0**	**19 895 204**	**0**	**33**	**0**	**269182**	**0**
S718C	10	**-9.09%**	41009	**-16.01%**	0	-	0	-	19	**+18.75%**	122309	**+14.51%**	2378	**-0.75%**	19 809 616	**-0.43%**	33	**0.0 %**	257499	**-4.34%**
P720R	10	**-9.09%**	43429	**-11.06%**	0	-	0	-	16	**0.0 %**	118264	**+10.72%**	2389	**-0.25%**	19 790 563	**-0.53%**	38	**+15.15%**	301926	**+12.16%**
P720Q	6	**-45.45%**	30170	**-38.21%**	0	-	0	-	20	**+25.00%**	124970	**+17.00%**	2426	**+1.25%**	19 670 064	**-1.13%**	35	**+6.06%**	268724	**-0.17%**
S719del	17	**+54.56%**	66538	**+36.27%**	0	-	0	-	19	**+18.75%**	128938	**+20.72%**	2392	**-0.17%**	19 758 723	**-0.69%**	35	**+6.06%**	287340	**+6.75%**
PD720-721R	12	**+9.09%**	61285	**+25.51%**	5	-	21737	-	17	**+6.25%**	111694	**+4.83%**	2424	**+1.17%**	20 056 467	**+0.81%**	30	**-9.09%**	223021	**-17.15%**
T739A	8	**-27.27%**	43316	**-11.29%**	0	-	0	-	17	**+6.25%**	112597	**+5.42%**	2414	**+0.75%**	19 945 540	**+0.25%**	36	**+9.09%**	269308	**+0.05%**
Mutants (mean)	10	**-9.09%**	47624	**-2.46%**	0.8	-	3623	-	18	**+12.50%**	119795	**+12.16%**	2404	**+0.33%**	19 838 496	**-0.29%**	34	**+3.03%**	267970	**-0.45%**

Supplemental Table 2: Time-resolved dynamics of DUB domain interactions with structural regions. "*N*" shows the number of interactions, and "*F*" indicates their frequency, with percentages showing the difference compared to the wild type. The table summarizes all interactions over a 100 ns simulation, including those exceeding 10% and 30% of the simulation time, highlighting the stability and prevalence of interactions across different regions.

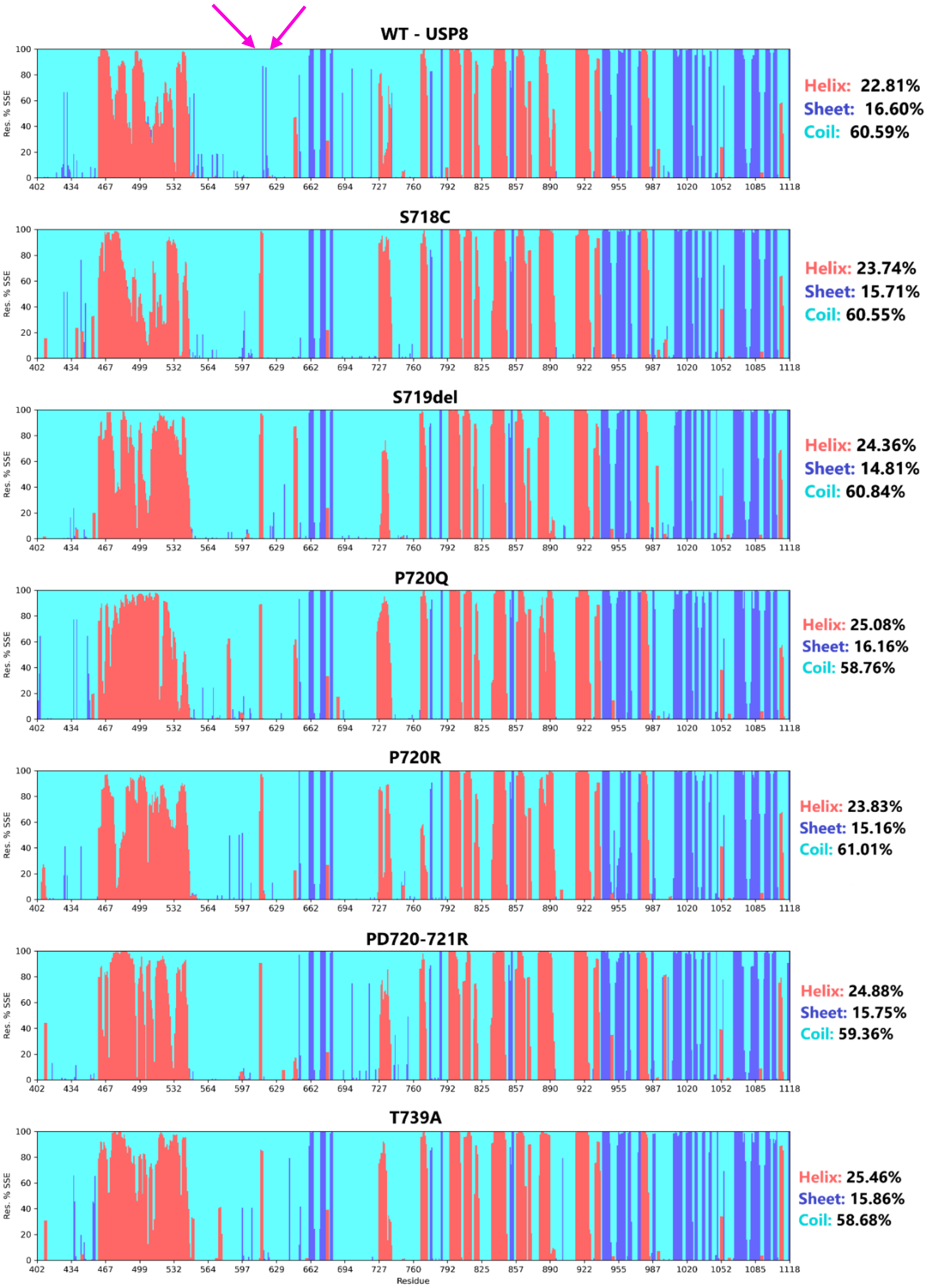

Supplemental Figure 3: Secondary Structure Composition Comparison: Pink arrows highlight the 610-615 region, showing a sheet in the wild type, replaced by a helix in all mutants

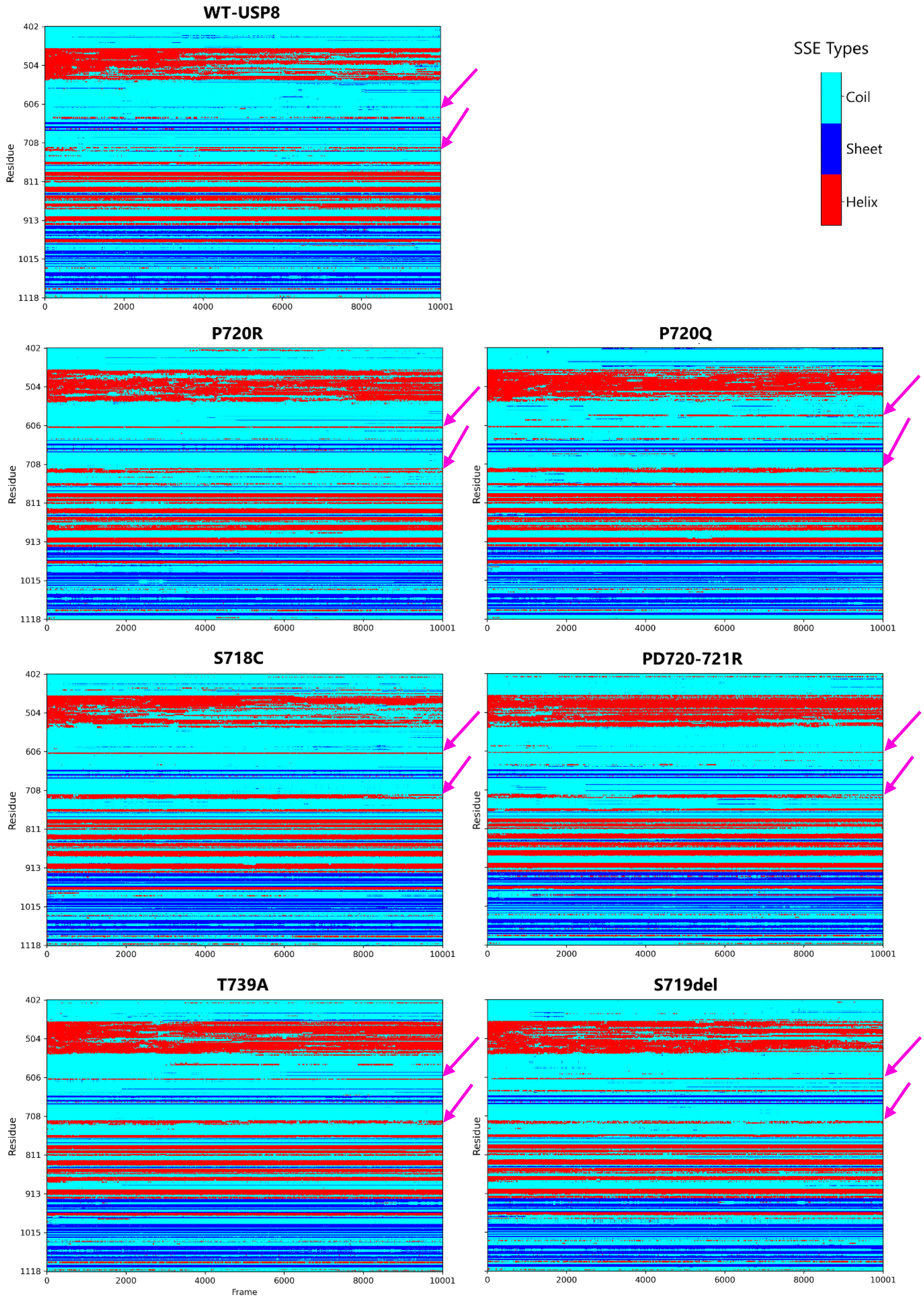

Supplemental Figure 4: Secondary Structure Dynamics Comparison Between WT and Mutant Variants Over Time Frames. Pink arrows indicate regions that differ between WT and are consistently observed across all six mutants.

	S718C	P720R	P720Q	S719del	PD720_721R	T739A
**LEU779 MET788**	Hydrophobic - 10	Hydrophobic - 1	Hydrophobic - 11	Hydrophobic - 1	Hydrophobic - 36	Hydrophobic - 1
**ASN818 ASN885**	VdWContact - 42	VdWContact - 3	HBDonor - 1<br>VdWContact - 78<br>HBAcceptor - 1	HBDonor - 1650<br>VdWContact - 2062<br>HBAcceptor - 1650	VdWContact - 690	HBDonor - 5112<br>VdWContact - 5797<br>HBAcceptor - 5112
**HIS822 THR853**	Hydrophobic – 318<br>VdWContact -144<br>HBDonor - 104<br>HBAcceptor -104	Hydrophobic - 1854<br>VdWContact - 254<br>HBDonor - 1996<br>HBAcceptor - 1996	Hydrophobic - 1316<br>HBDonor - 379<br>VdWContact - 637<br>HBAcceptor - 379	Hydrophobic - 560<br>HBDonor - 27<br>VdWContact - 122<br>HBAcceptor - 27	Hydrophobic - 1000<br>HBDonor - 96<br>VdWContact - 229<br>HBAcceptor - 96	Hydrophobic - 433<br>HBDonor - 30<br>VdWContact - 93<br>HBAcceptor - 30
**ASP883 GLU825**	HBDonor - 693<br>VdWContact - 1599<br>HBAcceptor - 693	HBDonor - 2390<br>VdWContact - 3944<br>HBAcceptor - 2390	HBDonor - 228<br>VdWContact - 778<br>HBAcceptor - 228	HBDonor - 8<br>VdWContact - 63<br>HBAcceptor - 8	HBDonor - 560<br>VdWContact - 1470<br>HBAcceptor - 560	HBDonor - 2<br>VdWContact - 38<br>HBAcceptor - 2
**GLN866 GLY1062**	HBDonor - 4710<br>HBAcceptor - 4710<br>VdWContact - 5358	HBDonor - 5094<br>HBAcceptor - 5094<br>VdWContact - 6135	HBDonor - 18<br>VdWContact - 55<br>HBAcceptor - 18	HBDonor - 68<br>VdWContact - 71<br>HBAcceptor - 68	HBDonor - 1571<br>VdWContact -1751<br>HBAcceptor - 1571	HBDonor - 4423<br>VdWContact - 5093<br>HBAcceptor - 4423
**ASP888 GLN916**	HBDonor - 14<br>VdWContact - 22<br>HBAcceptor - 14	HBAcceptor - 1<br>HBDonor - 8<br>VdWContact - 10	HBDonor - 19<br>VdWContact - 36<br>HBAcceptor - 19	HBDonor - 24<br>VdWContact - 24<br>HBAcceptor - 24	HBDonor - 3<br>VdWContact - 3<br>HBAcceptor - 3	HBDonor - 14<br>VdWContact - 19<br>HBAcceptor - 14
**ASN981 SER994**	HBDonor - 4304<br>HBAcceptor - 4304<br>VdWContact - 7179	HBAcceptor - 39<br>VdWContact - 863<br>HBDonor - 39	HBAcceptor - 4744<br>VdWContact - 6288<br>HBDonor - 4744	HBAcceptor - 941<br>VdWContact - 1611<br>HBDonor - 941	HBDonor - 2714<br>HBAcceptor - 2714<br>VdWContact - 4116	HBAcceptor - 1588<br>VdWContact - 4205<br>HBDonor - 1588
**ASP993 PHE983**	HBDonor - 1137<br>VdWContact - 9599<br>HBAcceptor - 1137	VdWContact - 798	HBDonor - 2<br>VdWContact - 7944<br>HBAcceptor - 2	VdWContact - 9955	VdWContact - 9970	VdWContact - 7979
**ARG989 ARG991**	HBAcceptor - 324<br>VdWContact - 622<br>HBDonor - 324	HBAcceptor - 4108<br>VdWContact 5187<br>HBDonor - 4108<br>Hydrophobic - 12	VdWContact - 1	VdWContact - 1	HBAcceptor - 107<br>VdWContact - 111<br>HBDonor - 107	HBAcceptor - 665<br>VdWContact - 1085<br>HBDonor - 665
**LEU792 MET834**	Hydrophobic - 102<br>VdWContact - 33	Hydrophobic - 81<br>VdWContact - 21	Hydrophobic - 11<br>VdWContact - 3	Hydrophobic - 2<br>VdWContact - 2	Hydrophobic - 2909<br>VdWContact - 851	Hydrophobic - 12<br>VdWContact - 1
**ASP979 LEU977**	VdWContact - 1	VdWContact - 58	Hydrophobic - 10<br>HBAcceptor - 1<br>VdWContact - 114<br>HBDonor - 1	VdWContact - 3	VdWContact - 5	VdWContact - 13
**THR978 TYR1016**	Hydrophobic - 198<br>VdWContact - 33	Hydrophobic - 66<br>VdWContact - 9	Hydrophobic - 318<br>VdWContact - 306<br>HBDonor - 92<br>HBAcceptor - 92	Hydrophobic - 3<br>VdWContact - 1	Hydrophobic - 1825<br>VdWContact - 690	Hydrophobic - 67<br>VdWContact - 75<br>HBDonor - 2<br>HBAcceptor - 2
**ASP979 TYR1016**	HBAcceptor - 110<br>VdWContact - 201<br>HBDonor - 110<br>Hydrophobic - 232	HBAcceptor - 1<br>VdWContact - 2<br>HBDonor - 1	Hydrophobic - 1173<br>VdWContact - 675<br>HBAcceptor - 158<br>HBDonor - 158	VdWContact - 1	Hydrophobic - 8<br>HBAcceptor - 12<br>HBDonor - 12<br>VdWContact - 40	Hydrophobic - 3414<br>VdWContact - 1203
**ASN897 GLU895**	HBAcceptor - 7<br>VdWContact - 9<br>HBDonor - 7	VdWContact - 4	VdWContact - 24<br>HBAcceptor - 1<br>HBDonor - 1	HBAcceptor - 667<br>VdWContact - 1069<br>HBDonor - 667<br>Hydrophobic - 33	Hydrophobic - 2<br>VdWContact - 3	HBAcceptor - 1<br>VdWContact - 1<br>HBDonor - 1
**SER1099 VAL1028**	HBDonor - 14<br>VdWContact - 90	HBDonor - 6<br>VdWContact - 19	HBDonor - 1<br>VdWContact - 5	HBDonor - 33<br>VdWContact - 51	HBDonor - 755<br>VdWContact - 800	HBDonor - 1<br>VdWContact - 1

Supplemental Table 3: Amino acid interaction pairs consistently found in all six mutant models but absent in the wild-type model. The types of interactions and their frequencies are presented as Interaction type - frequency (e.g., VdWContact - 800). The four most stable pairs are highlighted in red.

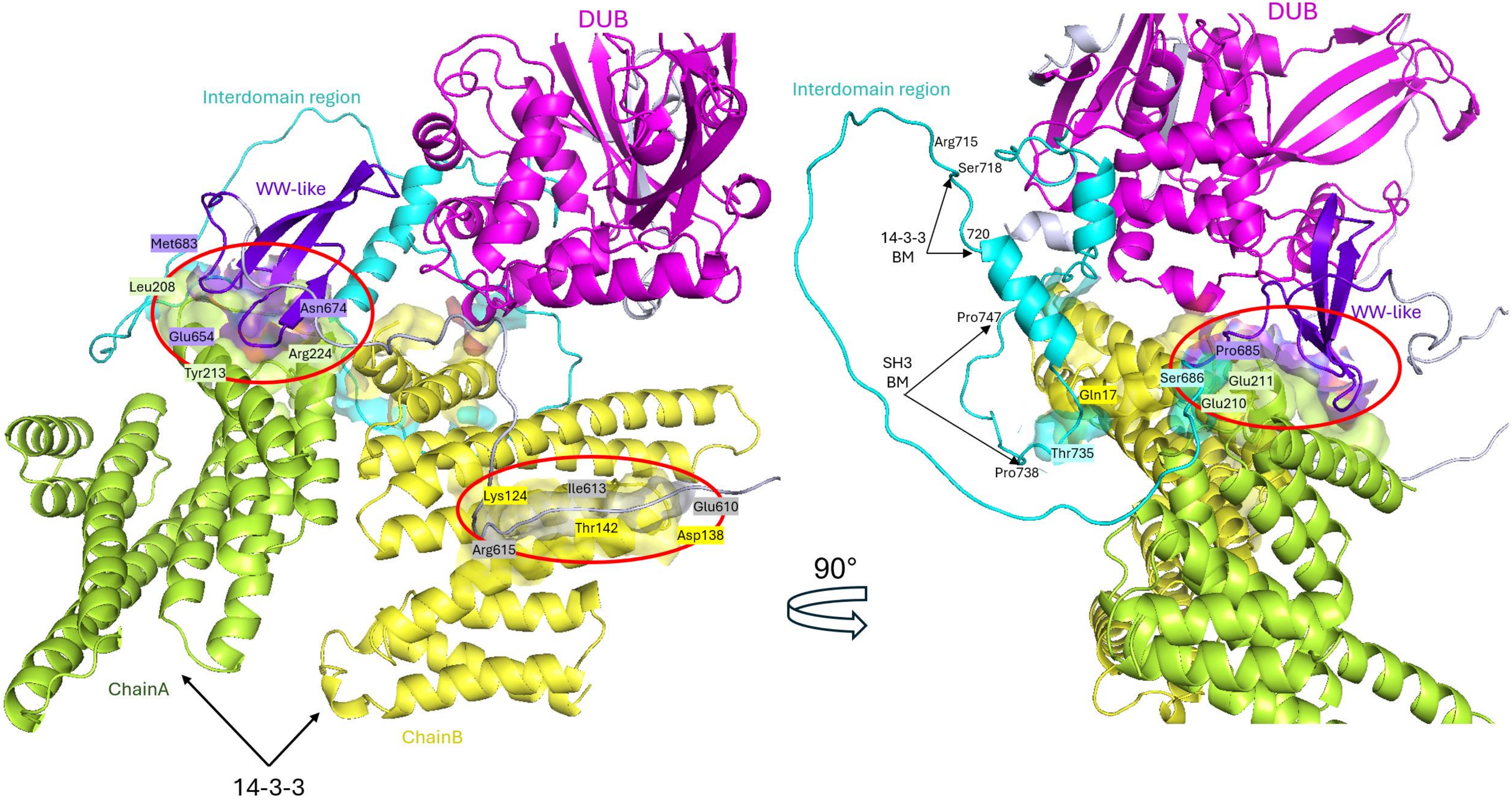

Supplemental Figure 5: Additional Protein-Protein Docking Analysis. Final frames from the simulations of all models were examined for potential binding to the 14-3-3 protein. The figure shows one of the complexes (mutant P720R + 14-3-3 β), illustrating a possible altered interaction between the unphosphorylated USP8 protein and the regulatory protein 14-3-3β.
